# Does the Goal Matter? Emotion Recognition Tasks Can Change the Social Value of Facial Mimicry Towards Artificial Agents

**DOI:** 10.3389/frobt.2021.699090

**Published:** 2021-11-17

**Authors:** Giulia Perugia, Maike Paetzel-Prüsmann, Isabelle Hupont, Giovanna Varni, Mohamed Chetouani, Christopher Edward Peters, Ginevra Castellano

**Affiliations:** ^1^ Human Technology Interaction Group, Eindhoven University of Technology, Eindhoven, Netherlands; ^2^ Uppsala Social Robotics Lab, Department of Information Technology, Uppsala University, Uppsala, Sweden; ^3^ Computational Linguistics, Department of Linguistics, University of Potsdam, Potsdam, Germany; ^4^ Joint Research Centre, European Commission, Seville, Spain; ^5^ LTCI, Télécom Paris, Institut Polytechnique de Paris, Paris, France; ^6^ Institut des Systèmes Intelligents et de Robotique, CNRS, Sorbonne Université, Paris, France; ^7^ KTH Royal Institute of Technology, Department of Computational Science Technology, Stockholm, Sweden

**Keywords:** human-robot interaction, human-agent interaction, affective computing, facial mimicry, anthropomorphism, uncanny valley, facial action coding system

## Abstract

In this paper, we present a study aimed at understanding whether the embodiment and humanlikeness of an artificial agent can affect people’s spontaneous and instructed mimicry of its facial expressions. The study followed a mixed experimental design and revolved around an emotion recognition task. Participants were randomly assigned to one level of humanlikeness (between-subject variable: humanlike, characterlike, or morph facial texture of the artificial agents) and observed the facial expressions displayed by three artificial agents differing in embodiment (within-subject variable: video-recorded robot, physical robot, and virtual agent) and a human (control). To study both spontaneous and instructed facial mimicry, we divided the experimental sessions into two phases. In the first phase, we asked participants to observe and recognize the emotions displayed by the agents. In the second phase, we asked them to look at the agents’ facial expressions, replicate their dynamics as closely as possible, and then identify the observed emotions. In both cases, we assessed participants’ facial expressions with an automated Action Unit (AU) intensity detector. Contrary to our hypotheses, our results disclose that the agent that was perceived as the least uncanny, and most anthropomorphic, likable, and co-present, was the one spontaneously mimicked the least. Moreover, they show that instructed facial mimicry negatively predicts spontaneous facial mimicry. Further exploratory analyses revealed that spontaneous facial mimicry appeared when participants were less certain of the emotion they recognized. Hence, we postulate that an emotion recognition goal can flip the social value of facial mimicry as it transforms a likable artificial agent into a distractor. Further work is needed to corroborate this hypothesis. Nevertheless, our findings shed light on the functioning of human-agent and human-robot mimicry in emotion recognition tasks and help us to unravel the relationship between facial mimicry, liking, and rapport.

## 1 Introduction

The success of artificial agents in areas like healthcare, personal assistance, and education highly depends on whether people perceive them as likable and pleasant to interact with. In the lab, people’s perceptions of an artificial agent can be easily measured with questionnaires and interviews. In real-life settings, instead, the artificial agent is on its own and the explicit evaluation of the interaction is not always feasible. In fact, in these contexts, people might skip the proposed surveys or reply carelessly due to lack of time and interest (Chung and Cakmak, 2018). A more promising approach in such contexts may be the use of behavioral measures. While behavioral measures are in general extensively used in Human-Agent and Human-Robot Interaction (HAI and HRI), they are seldom linked to people’s self-reported perceptions (e.g., likability and engagement, see Perugia et al., 2020a; Perugia et al., 2021). In this paper, we focus on facial mimicry—the mirroring of another person’s facial expressions (Hatfield et al., 1992)—and aim to provide a fundamental understanding of *whether humans mimic the facial expressions of the six basic emotions displayed by artificial agents and whether facial mimicry is linked to people’s perception of artificial agents*. To the best of our knoweldge, no previous work in HAI and HRI has focused on this goal, hence this paper constitutes one of the first attempts to understand whether facial mimicry of the six basic emotions is related to people’s perceptions of artificial agents.

From psychology, we know that facial mimicry increases with rapport (Tickle-Degnen and Rosenthal, 1990; Hess et al., 1995), but also appears in first acquaintances between individuals as a sign of liking (Chartrand and Bargh, 1999; Kulesza et al., 2015). Studies on facial mimicry in HAI and HRI have so far mostly focused on whether artificial agents are liked better when given the ability to mimic a human interaction partner (Hoegen et al., 2018; Numata et al., 2020; Riek et al., 2009). Hofree et al. (2014) were among the few researchers who investigated whether human interaction partners mimic the facial expressions of artificial agents as well. In their study, they disclosed that people’s mimicry of an android’s facial expressions of anger and happiness is connected with their perceptions of the agent’s humanlikeness only when the android is co-present. In our study, we extend Hofree et al. (2014)’s work by 1) including a *wider spectrum of artificial agents*, 2) employing an overall *less realistic humanoid robot that allows for easy alteration of facial cues* (i.e., Furhat), 3) focusing on *all six basic emotions*, and 4) using a *computer vision technique* in lieu of Electromyography (EMG) to estimate people’s facial mimicry. With respect to EMG, computer vision is far less obtrusive and hence more viable for field use.

In this study, we involved 45 participants in an emotion recognition task with three artificial agents varying in embodiment (i.e., physical Furhat robot, video-recorded Furhat robot, and a virtual agent) and humanlikeness (i.e., humanlike, characterlike, and morph). Moreover, we also included a human control condition to understand differences between mimicry of artificial and natural agents. The emotion recognition task used in our experiment was divided into two phases. In the first phase, participants were asked to observe the facial expressions of the six basic emotions as expressed by the three artificial agents and a video-recorded human (i.e., the control), and pick the correct one from a list. In the second phase, instead, they were asked to observe the same facial expressions in re-shuffled order, mimic their temporal dynamics as closely as possible, and only afterwards recognize them. Based on Kulesza et al. (2015), this latter phase was carried out under the pretense that intentional mimicry of facial expressions could actually improve participants’ emotion recognition. Participants’ faces were video-recorded in both stages of the experiment and the activation of the action units (AU) corresponding to the six basic emotions was determined through Hupont and Chetouani (2019)’s AU intensity detector. In the first part of the study, we gauged which facial expressions were *spontaneously* mimicked by participants. In the second part of the study, we focused instead on participants’ *instructed* mimicry, and estimated how accurate participants were in replicating the temporal dynamics of the observed facial expressions.

The aim of this study is to understand 1) whether an artificial agent’s embodiment and humanlikeness can influence people’s spontaneous and instructed facial mimicry (as suggested by Hofree et al., 2014 and Mattheij et al., 2013; Mattheij et al., 2015), 2) if spontaneous facial mimicry is related to people’s perceptions of artificial agents, especially in terms of anthropomorphism, social presence, likability, and uncanniness (perceptual dimensions expected to be influenced by the agent’s level of humanlikeness), and 3) whether there is a link between instructed and spontaneous facial mimicry. The overarching ambition of this work is to explore *whether spontaneous facial mimicry can be used as an implicit, unconscious cue of liking and rapport in HAI and HRI, and whether instructed facial mimicry can act as its proxy in settings where spontaneous facial mimicry is difficult to gauge*. Our work contributes to efforts paving the way towards unobtrusive automatic assessment of facial mimicry in interactions with artificial agents, hence facilitating the measurement of liking and rapport through behavioral cues in the future.

## 2 Related Work

Facial mimicry is the spontaneous imitation of another individual’s facial expression without explicit instruction to do so (Hatfield et al., 1992). Within the area of facial mimicry research, emotional mimicry refers to the spontaneous mirroring of a facial expression with inherent emotional meaning, for instance, wincing when observing others in pain (Bavelas et al., 1986) or frowning at another person’s frown. This paper focuses on people’s mimicry of the six basic emotions—happiness, sadness, surprise, anger, fear, and disgust (Ekman and Rosenberg, 1997)—as displayed through the facial expressions of artificial and natural agents. Within subsections 2.1, 2.2, 2.3, we give an account of the different theories on the nature and functioning of spontaneous facial mimicry in human-human interactions (HHI). We then describe the literature on human-agent and human-robot facial mimicry in subsection 2.4, and explain our interest in instructed facial mimicry in subsection 2.5. Since the extant HAI and HRI literature has hardly ever explored the relationship between facial mimicry and people’s perceptions of artificial agents, in subsection 2.4, 2.5, we mostly present studies focused on changes in facial mimicry due to an agent’s humanlikeness and co-presence.

### 2.1 Nature of Spontaneous Facial Mimicry

There are two main theoretical perspectives on the nature and functioning of emotional mimicry: a motor and an emotional perspective. The *motor perspective* holds that emotional mimicry is an unconscious, unintentional, unemotional, and reflex-like matching of observed facial expressions (Chartrand and Bargh, 1999). Within this context, the *associative sequence learning* (ASL) approach posits that mimicry happens by virtue of a learned long-term association between an action stimulus (e.g., a person’s smile) and an action response (e.g., the observer’s smile; Heyes (2011), which holds as long as the action stimulus (e.g., the observed facial expression) is similar to other stimuli previously associated with a certain motor action (e.g., the observer’s facial expression).

Another theoretical formalization within the motor perspective is the *automatic embodiment account*, which postulates that mimicry is the embodied motor simulation of an observed emotion that serves the purpose of emotion recognition (Niedenthal et al., 2010) According to this approach, we mimic another individual’s facial expressions to better recognize and differentiate them.

As opposed to the motor perspective, the *emotional perspective* sees mimicry as a marker of subtle affective states arising in response to emotional stimuli (Dimberg, 1990; Dimberg, 1997). Within this perspective, the *facial-feedback hypothesis* (Tomkins, 1984; Izard, 1977), which dates back to Darwin (Darwin and Prodger, 1998), posits that “the sight of a face that is happy, loving, angry, sad, or fearful (…) can cause the viewer to mimic elements of that face and, consequently, to catch the other’s emotions” (Hatfield et al., 1992). With a slightly different line of thought, the *affect-matching account* suggests that observing a facial expression triggers a corresponding affective state in the observer, which *then* generates the mimicking act (Dimberg et al., 2000). Within the emotional perspective, there is hence no clear consensus yet as to whether the affective state arising from an emotional stimulus precedes or succeeds mimicry.

The motor and emotional perspectives make somewhat different claims on the outcomes of emotional mimicry (Moody et al., 2007). The motor perspective assumes that facial mimicry is always consistent with the observed facial expression (i.e., emotion-congruent mimicry). For instance, an expression of anger can only trigger a corresponding expression of anger. On the opposite, the emotional perspective suggests that mimicry is related to the action tendencies associated with a stimulus (e.g., competitive and collaborative tasks, Lanzetta and Englis, 1989). Thus an expression of anger can trigger anger but also fear (i.e., valence-congruent mimicry), and the type of emotion triggered depends on the meaning associated with the observed facial expression and the context where mimicry takes place (Fischer et al., 2012).

### 2.2 Evidence Supporting Theoretical Accounts on Spontaneous Facial Mimicry

In general, there is little experimental support for the motor perspective. Available studies almost exclusively focused on facial mimicry of happiness and anger. As Hess and Fischer (2013, 2014) underline, such studies only confirm that people display a valence-congruent facial expression when exposed to happiness and anger (i.e., smiling to happiness, frowning to anger). However, they do not fully back up emotion-congruent facial mimicry, which is at the core of the motor perspective. With regards to the automatic embodiment account, several studies have investigated whether blocking facial mimicry impairs the correct recognition of emotional facial expressions (Niedenthal et al., 2001; Hawk et al., 2012). Current evidence supports this position only partially. Indeed, mimicry seems to be crucial for emotion recognition but only when it comes to recognizing ambiguous or subtle facial expressions (Hess and Blairy, 2001; Fischer et al., 2012).

There are a number of studies that support the emotional perspective. For instance, Laird and Bresler (1992) noticed that when people are asked to reproduce facial expressions of fear, anger, sadness, and disgust, they also report experiencing those emotions. Moreover, Ekman et al. (1983) note that the muscular reproduction of the facial expressions of the six basic emotions activates the Autonomic Nervous System (ANS) in a similar way as to when people actually experience those emotions. Finally, Dimberg and Thunberg (1998) describe how the facial response system that is responsible for mimicry responds to emotions faster (300–400 ms) than the ANS (1–3 s), thus finding support for the affect-matching account. Further support for the emotional perspective was also brought by Moody et al. (2007) who found that fear priming elicits expressions of fear in response to both fear and anger, thus demonstrating that mimicry is not a purely automatic mirroring of an observed emotion, but has an intrinsic emotional meaning.

### 2.3 The Social Value of Spontaneous Facial Mimicry

Regardless of their different views on the nature of facial mimicry, both the motor and the emotional perspective posit that facial mimicry serves a social purpose. In one case (i.e., motor perspective), it serves to recognize and respond to other people’s emotions. In the other case (i.e., emotional perspective), it serves the purpose of emotional contagion (Hatfield et al., 1993; Varni et al., 2017), as to say “the tendency to automatically mimic and synchronize movements, expressions, postures, and vocalizations with those of another person and, consequently, to converge emotionally” (Hatfield et al., 1992). The literature suggests that mimicry is indicative of higher liking during first acquaintances (Chartrand and Bargh, 1999; Kulesza et al., 2015; Calvo-Barajas et al., 2020), stronger rapport in already established relationships (Hess et al., 1995) and that it increases when two interaction partners are given the goal to affiliate (Lakin et al., 2003). In fact, Hess et al. (1995) found that watching funny movies with friends elicits more laughs than watching them with strangers. Consistently, Fischer et al. (2012) discovered that dyads of friends mimic each other’s smiles of pride more than strangers do. Hess and Fischer (2013) and Bourgeois and Hess (2008) propose that mimicry acts as a *social regulator* as it communicates the intention to bond. Since emotional mimicry is known to be related with interpersonal stance (Prepin et al., 2013), social tuning (Bernieri, 1988), bonding (Jaques et al., 2016), and rapport (Tickle-Degnen and Rosenthal, 1990; Gratch et al., 2006; Wang and Gratch, 2009), we consider it an important phenomenon to study in Human-Robot (HRI) and Human-Agent Interaction (HAI). In fact, if facial mimicry was found to work similarly for artificial agents and humans, it could inform future work investigating its use as an implicit and unconscious measure of the quality of interaction in HAI and HRI (Perugia et al., 2020b).

### 2.4 Spontaneous Facial Mimicry of Virtual Agents and Social Robots

In face-to-face interactions between humans, acted facial expressions constitute the only possibility of studying spontaneous facial mimicry in a controlled way. However, acted facial expressions can be perceived by humans as being inauthentic and hence might hinder the occurrence of mimicry. For this reason, in psychology, studies on spontaneous facial mimicry have almost exclusively focused on static images or videos of facial expressions, with these latter being sometimes used to simulate live video-sessions (Kulesza et al., 2015). With respect to humans, virtual and robotic agents give the unique possibility to investigate spontaneous mimicry in face-to-face interactions occurring in real-time while preserving control over the experimental setup (Hoegen et al., 2018). This is because they enable researchers to manipulate only a few facial action units (AU) and control their activation over time. In this sense, the use of virtual and robotic agents not only allows to investigate whether spontaneous facial mimicry occurs or not in specific contexts, but also opens up the possibility to understand whether its temporal dynamics are replicated.

While human-agent mimicry has been explored more thoroughly (Gratch et al., 2006; Hoegen et al., 2018), studies on human-robot mimicry gained popularity more recently. Such a delay is probably due to the fact that robots’ faces were not provided with enough degrees of freedom to accurately reproduce facial expressions until very recently. Most available studies on human-robot and human-agent mimicry focus on endowing agents with the ability to mimic the facial expressions of human interactants and observing how this ability affects people’s perceptions and reactions (Hoegen et al., 2018; Numata et al., 2020; Riek et al., 2009). Only a few studies investigate people’s spontaneous mimicry of an artificial agent’s facial expressions, and, to the best of our knowledge, only one of these discussed the relationship between facial mimicry and people’s perceptions of a robot (i.e., humanlikeness (Hofree et al., 2014).

The available studies in HAI and HRI show similar results to human-human mimicry, with the main difference residing in the lower intensity and slower speed of human-agent and human-robot mimicry. For instance, Mattheij et al. (2015, 2013) found evidence for the spontaneous mimicry of happiness, surprise, and disgust in the context of HAI and Philip et al. (2018) disclosed that people spontaneously mimic virtual agents’ facial expressions of joy, anger, and sadness. They also observed that mimicry is less intense when it is directed to a virtual agent with respect to a human one. Similarly, in HRI, Hofree et al. (2014) observed that people mimic a video-recorded android (i.e., Hanson’s Einstein robot) to a lesser extent than a video-recorded human. Furthermore, they discovered that, while the facial expressions of a video-recorded android are mimicked only when the robot is perceived as highly humanlike, physically co-present androids are mimicked regardless of the perceptions they elicit. Hence, they proposed that it is the robot’s co-presence that makes its humanlike appearance highly salient, and in turn elicits spontaneous facial mimicry. Following this line of thought, in the present study, *we manipulated the artificial agents’ humanlikeness, as well as their embodiment, and attempted to understand whether these influenced spontaneous facial mimicry.* We employed all three embodiments used by Hofree et al. (2014)—a video-recorded human, a video-recorded robot, and a physical robot. Moreover, we added a virtual agent as in Mattheij et al. (2013, 2015). In line with Li (2015), we considered: 1) the video-recorded robot as *artificial, physically embodied*, but *not co-presen*t; 2) the physical robot as *artificial, physically embodied*, and *co-present*; and 3) the video-recorded human as *natural, physically embodied*, but *not co-present*. While Li (2015) differentiates between *physical* and *digital co-presence*, in this work we combined the two into one single category of *co-presence* to distinguish between the two video-recordings that capture behavior of the past and hence do not share the same environment and time with the participant (i.e., video-recorded robot and video-recorded human) from the virtual agent which shares the same environment and time with the participant. Consequently, we categorize the virtual agent as *artificial, virtually embodied*, and *co-present*.

In HHI, Bourgeois and Hess (2008) showed that the social context in which the interaction takes place has the power to influence emotional mimicry. While happy expressions are mimicked regardless of whether an observed person is an in-group or out-group member, expressions of sadness are mimicked only between in-group members. Likewise, in HRI, Hofree et al. (2018) showed that participants mimicked a robot’s smiles and frowns when cooperating with it, but displayed inverse mimicry (i.e., frowned at the robot’s smiles and smiled in response to its frowns), when the context was competitive. To circumvent this problem, in this study, we showed the agents’ facial expressions to participants in a non-interactive context inspired by Kulesza et al. (2015). Similar to Hofree et al. (2014), in this study, *we asked participants to carefully observe the agents’ facial expressions*. Inspired by Kulesza et al. (2015), however, *we also gave them the goal to recognize the emotion displayed by the agent*.

### 2.5 Spontaneous and Instructed Facial Mimicry

Facial mimicry can further be divided into spontaneous and instructed. Spontaneous facial mimicry, which we have discussed so far, occurs unconsciously, without any specific instruction (Hatfield et al., 1992). Instructed facial mimicry, instead, is deliberate mimicry of facial expressions that occurs consciously as a result of specific instructions (McIntosh et al., 2006; Paetzel et al., 2017). In their study, Hofree et al. (2014) used instructed facial mimicry to ensure that the facial expressions of the android they used were visible, feasible to imitate, and that electromyography (EMG) was working properly. Interestingly, they reported similar results for spontaneous and instructed facial mimicry. In fact, similar to spontaneous facial mimicry, the instructed facial mimicry of the video-recorded android was less intense than the one directed to the video-recorded human. This result brought us to hypothesize that instructed facial mimicry might be somehow linked to spontaneous facial mimicry. To deepen our understanding of the relationship between instructed and spontaneous facial mimicry, in this paper, *we explore whether spontaneous facial mimicry can be predicted by people’s ability to accurately reproduce the dynamics of an agent’s facial expressions of the six basic emotions upon instruction to do so*. Moreover, *we study whether artificial agents’ embodiment and level of humanlikeness can affect instructed facial mimicry in a way that is analogous to spontaneous facial mimicry*. Should instructed facial mimicry be found to significantly predict spontaneous facial mimicry, it could be used as an *explicit* cue of people’s social tuning with an artificial agent and could act as proxy of spontaneous facial mimicry.

## 3 Research Questions and Hypotheses

In this work, we explore the influence of embodiment and humanlikeness on people’s spontaneous and instructed mimicry of artificial agents’ facial expressions of the six basic emotions. Based on Hofree et al. (2014) and Mattheij et al. (2013, 2015), we chose three artificial embodiments for this study: a video-recorded robot, a physical robot, and a virtual agent. Furthermore, we added a control condition in which participants observed the facial expressions of a video-recorded human. To change the artificial agents’ level of humanlikenss, we manipulated their facial features to resemble those of a characterlike face, a humanlike face, and a face that includes features from both of them (i.e., a morph). Humanlikeness was chosen as an independent variable in our study not only because Hofree et al. (2014) found it to be salient for facial mimicry, but also since it is known to influence people’s perceptions of an agent’s anthropomorphism, social presence, and uncanniness (Mori et al., 2012; Kätsyri et al., 2015), which are perceptual dimensions that in turn affect liking and rapport. Our first group of research questions (RQ1a—RQ1c) concerns spontaneous facial mimicry:

(RQ1a) To what extent does the humanlikeness of artificial agents influence people’s spontaneous facial mimicry?

(RQ1b) To what extent does the embodiment of artificial agents influence people’s spontaneous facial mimicry?

(RQ1c) Does spontaneous facial mimicry differ between artificial and human agents?

Our second group of research questions (RQ2a—RQ2c) revolves around instructed facial mimicry. In previous work (Paetzel et al., 2017), we investigated how well people were able to reproduce the dynamics of a laughter performed by an artificial agent that they were explicitly instructed to mimic. In this paper we focus on facial expressions of the six basic emotions instead. Here, we aim to understand whether the agents’ embodiment and humanlikeness can affect instructed facial mimicry similar to how they affect spontaneous facial mimicry. Therefore, we pose the following research questions:

(RQ2a) To what extent does the humanlikeness of artificial agents influence people’s ability to mimic their facial expressions as accurately as possible when instructed to do so?

(RQ2b) To what extent does the embodiment of artificial agents influence people’s ability to mimic their facial expressions as accurately as possible when instructed to do so?

(RQ2c) Does instructed facial mimicry differ between artificial and human agents?

The long-term goal of our research is to provide initial insights on the development of implicit and explicit behavioral measures that can extend or replace questionnaire-based investigations of the perception of artificial agents. Previous work in human-human interaction has highlighted that spontaneous facial mimicry signals liking in first acquaintances (Chartrand and Bargh, 1999; Kulesza et al., 2015) and rapport in established relationships (Hess et al., 1995; Fischer et al., 2012). Liking and rapport are complex constructs known to be influenced by factors such as the appearance and embodiment of an agent (Perugia et al., 2021; Paetzel et al., 2020; Paetzel-Prüsmann et al., 2021). In this study, besides understanding the role of embodiment and humanlikeness in facial mimicry, we aim to gain more insights on the relationship between spontaneous facial mimicry and a few of the perceptual dimensions known to influence rapport and liking. The relationship between facial mimicry and people’s perceptions of artificial agents has been addressed only seldom in the HAI and HRI literature.

(RQ3) To what extent can spontaneous facial mimicry predict the agent’s perceived social presence, anthropomorphism, uncanniness, and likability?

From the related literature, we know that the occurrence of spontaneous facial mimicry can be an important predictor of the rapport people build with a human or artificial interaction partner. However, due to occlusions of the face and the subtlety of the mimicked facial expressions, it is often difficult to capture and quantify spontaneous facial mimicry in natural settings and more complex interactions. In these contexts, instructed facial mimicry could act as a proxy of spontaneous facial mimicry and could be used in place of a questionnaire as an *explicit* indirect cue of liking and rapport. Our fourth research question is thus concerned with the relation between instructed and spontaneous facial mimicry:

(RQ4) To what extent does instructed facial mimicry predict spontaneous facial mimicry?

Based on related studies performed by Hofree et al. (2014), Chartrand and Bargh (1999), Kulesza et al. (2015), and Hess et al. (1995), we expected that:

H1) Physically embodied, co-present, humanlike artificial agents elicit higher spontaneous facial mimicry with respect to virtually embodied, non-co-present, non-humanlike artificial agents.
H2) Physically embodied, co-present, humanlike artificial agents elicit higher instructed facial mimicry with respect to virtually embodied, non-co-present, non-humanlike artificial agents.
H3) Spontaneous facial mimicry positively predicts people’s evaluations of the agents’ anthropomorphism, social presence, and likability, and negatively predicts their perceived uncanniness.
H4) Instructed facial mimicry positively predicts spontaneous facial mimicry.

## 4 Methodology

**FIGURE 1:**
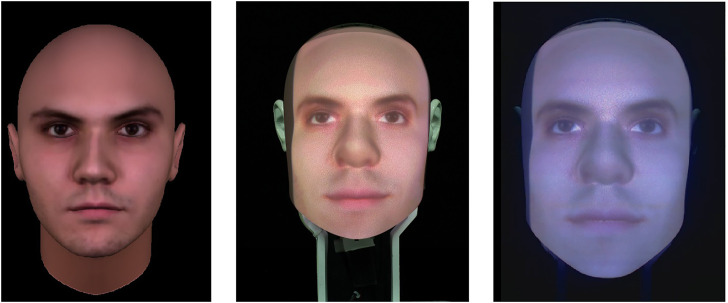
Different types of artificial embodiments used in the experiment. From left to right: a virtual agent; a physical Furhat robot; and a video recording of the Furhat robot.

**FIGURE 2:**
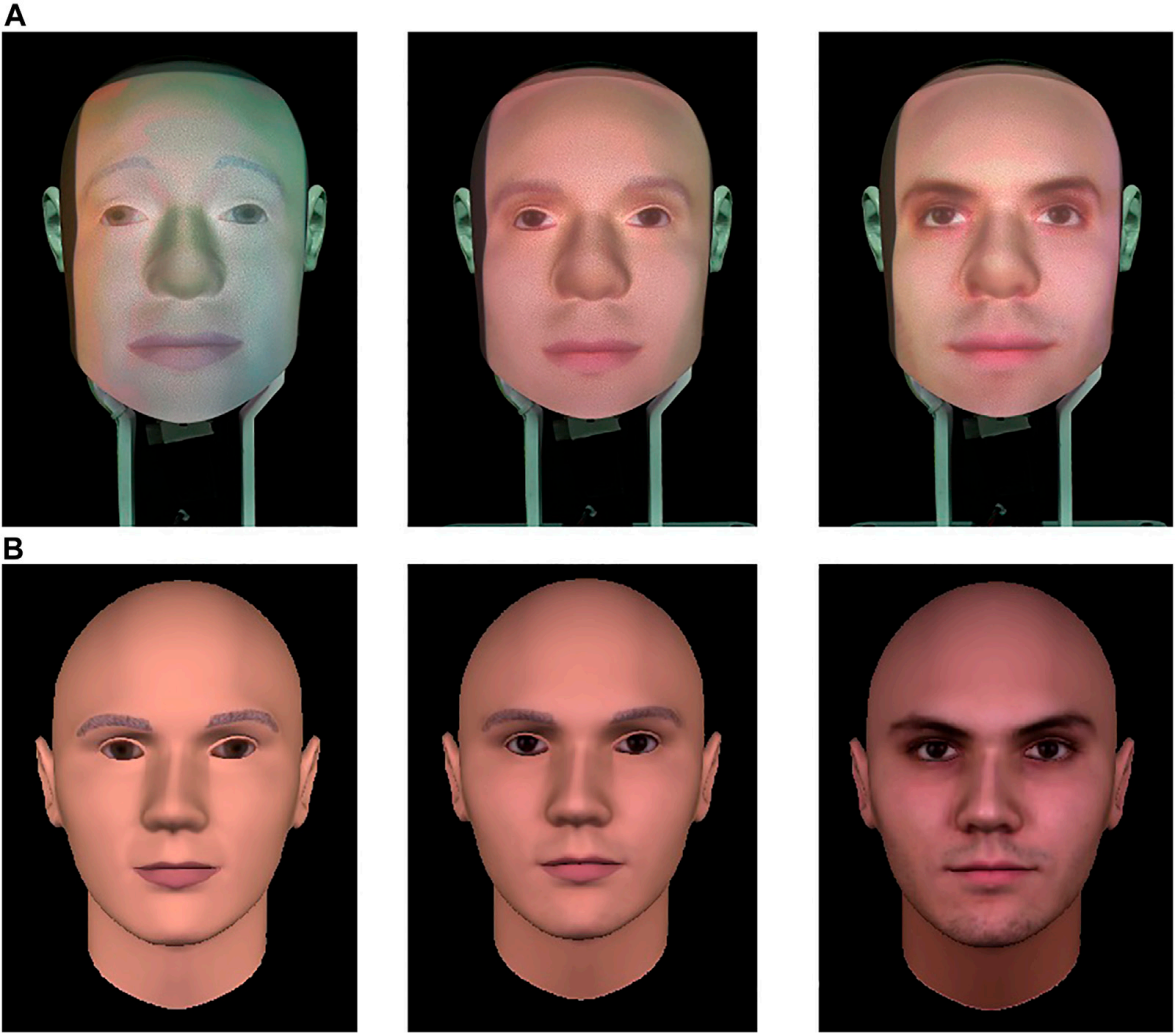
Levels of humanlikeness used in the experiment in the Furhat robot **(A)** and virtual agent **(B)**. Left: characterlike; right: humanlike; center: morph.

Our study followed a 3 x 3 + 1 mixed experimental design with:

• *Embodiment* as within-subject variable with three types of artificial embodiment: a virtual agent, a physical Furhat robot (Al Moubayed et al., 2012), and a video-recording of the Furhat robot (cf. Figure 1)
• *Humanlikeness* as between-subject variable with three levels of humanlikeness: *humanlike*, *characterlike* and a *morph* between the humanlike and the characterlike (cf. Figure 2)

Furthermore, we included a control condition (+1) for the embodiment in which participants observed a video-recorded human (cf. Figure 1). This control condition was the same across all levels of the agent’s humanlikeness.

The experimental design was informed by Kulesza et al. (2015) and consisted of two parts. In the first part, each participant was asked to observe the facial expressions of the agents and identify which of the six basic emotions they displayed (i.e., happiness, sadness, surprise, anger, fear, and disgust). In the second part, which occurred after a 5-min break, participants were explicitly told that the accuracy of mimicry could improve emotion recognition. Consequently, they were instructed to observe the facial expressions corresponding to the six basic emotions performed by the same agents (in randomized order), mimic them as closely as possible, and identify them only after they finished mimicking. The first part of the experiment allowed us to study spontaneous facial mimicry, the second part to investigate instructed facial mimicry. Participants were video-recorded during both parts of the study.

**FIGURE 3:**
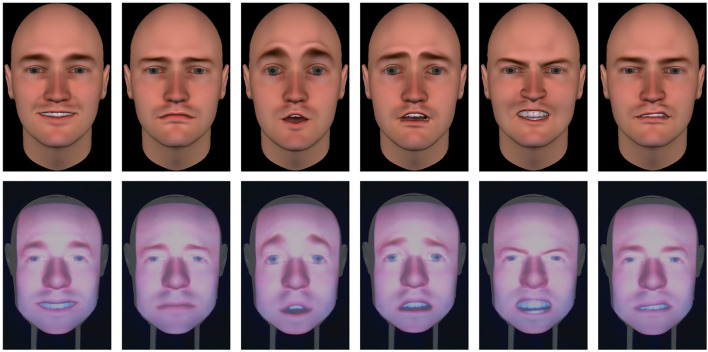
Facial expressions of the six basic emotions. From left to right: happiness, sadness, surprise, fear, anger and disgust. From top to bottom: virtual agent, Furhat robot.

Each participant observed a set of facial expressions performed by the video-recorded human and the three artificial agents. All three artificial agents had the same level of humanlikeness but differed in their embodiment. Each set of facial expressions was composed of expressions of the six basic emotions performed twice by each agent. Within each set, the order of presentation of the stimuli was randomized, and no two facial expressions of the same type occurred one after the other. The order of presentation of the artificial and human agents was shuffled using Latin Squares. In total, each participant observed 48 facial expressions for each part of the study. Emotional facial expressions were presented in short sequences of 5 s including onset, apex and offset, without vocalizations nor head movements (cf. Figure 3).

### 4.1 Participants

An a priori sample size calculation for a repeated measures ANOVA with within-between interactions (3 x 3), medium effect size (0.25), *α* error probability of 0.05, and Power of 0.9 revealed a required total sample size of 45. We hence recruited 46 participants from an international study program in Computer Science at Uppsala University. Participants had at least a high school degree and came from a diverse geographic background (44.4% Swedish). The 46 participants were randomly allocated to the three conditions corresponding to the different levels of humanlikeness of the artificial agents: *characterlike* (N = 15; 11 male; 4 female; 0 other/prefer not to say), *humanlike* (N = 16; 13 male; 3 female; 0 other/prefer not to say), and *morph* (N = 15; 12 male; 3 female; 0 other/prefer not to say). Due to a misunderstanding of the study task, we excluded the data of one male participant from the humanlike condition. The final sample of participants had a mean age of 26.16 years (*SD* = 4.37) and was composed of 10 people identifying themselves a female and 35 as male. None of the participants had previously interacted with the Furhat robot.

### 4.2 Embodiment and Humanlikeness

As a robot, we chose the Furhat platform (Al Moubayed et al., 2012). Furhat is a blended robot head consisting of a rigid mask on which a facial texture is projected from within. We chose the Furhat robot for this experiment as its virtual face allowed us to easily alter facial features and design smooth and noiseless facial expressions.

We designed three different facial textures for the artificial agents. The humanlike face was created from pictures of a real human face using the FaceGen Modeller^1^. The characterlike face was the standard Furhat face with sketched “drawing-like” lips and eyebrows. Finally, the morph face was created by blending the humanlike and characterlike skin textures in the Paint.NET digital photo editing package. The three different textures we applied to the artificial agents were selected from a set of 28 faces tested in a pre-study on Amazon Mechanical Turk (AMT). Since initial experiments with the Furhat robot found the face mask without any projection to be perceived as male and dominant (Paetzel et al., 2016), we limited the set of stimuli to male faces. The same texture we used for the Furhat robot was also utilized to create the virtual agent’s face. The video-recorded robot was obtained by recording the physical Furhat. For the human control condition, instead, we selected the video-recordings of a male person from the MUG database (Aifanti et al., 2010).

### 4.3 Synthesis of Facial Expressions

The human in the MUG database was video-recorded while performing the facial expressions of the six basic emotions following the Facial Action Coding System FACS, (Hager et al., 2002; Ekman et al., 2002) and an onset-apex-offset temporal scheme. We designed the facial expressions of the artificial agents by replicating the dynamics of the human video recording as closely as possible. Unfortunately, as in Furhat’s IrisTK animation system (Skantze and Al Moubayed, 2012), some facial Action Units (AUs) are combined and cannot be controlled separately, the facial expressions of the human and those of the artificial agents slightly differed (cf. Figure 3). An expert trained in the FACS ensured that the final set of stimuli for the artificial agents was still following the FACS′ guidelines.

We conducted an online preliminary study with 60 participants recruited on Amazon Mechanical Turk to assess whether the naturalness, recognition rate, and intensity of the facial expressions of the six basic emotions we designed was comparable across artificial agents. Crowd-workers were shown videos of the facial expressions of the six basic emotions plus a neutral expression displayed by either the virtual agent, the Furhat robot, or a human control (between-subjects). Our validation study revealed that the facial expressions of the two artificial agents were comparable, except for a difference in sadness. As expected given the limitations of the IrisTK animation system, the expression of anger displayed by the human control was rated as less intense compared to the one displayed by the artificial agents, and the expression of disgust displayed by the human control was rated as more intense compared to the one displayed by the artificial agents. With regards to naturalness, the expression of happiness was perceived as more natural for the human control than for the artificial agents, and the human video received a higher rating in naturalness than the videos of the artificial agents. We can hence conclude that, while some differences between the embodiments prevail, they mostly concern differences between the human control vs the artificial agents. Since the human video was only used as control in our study, and most of the analyses involved comparisons between artificial agents, we did not think these differences could affect the results.

### 4.4 Experimental Setup

**FIGURE 4:**
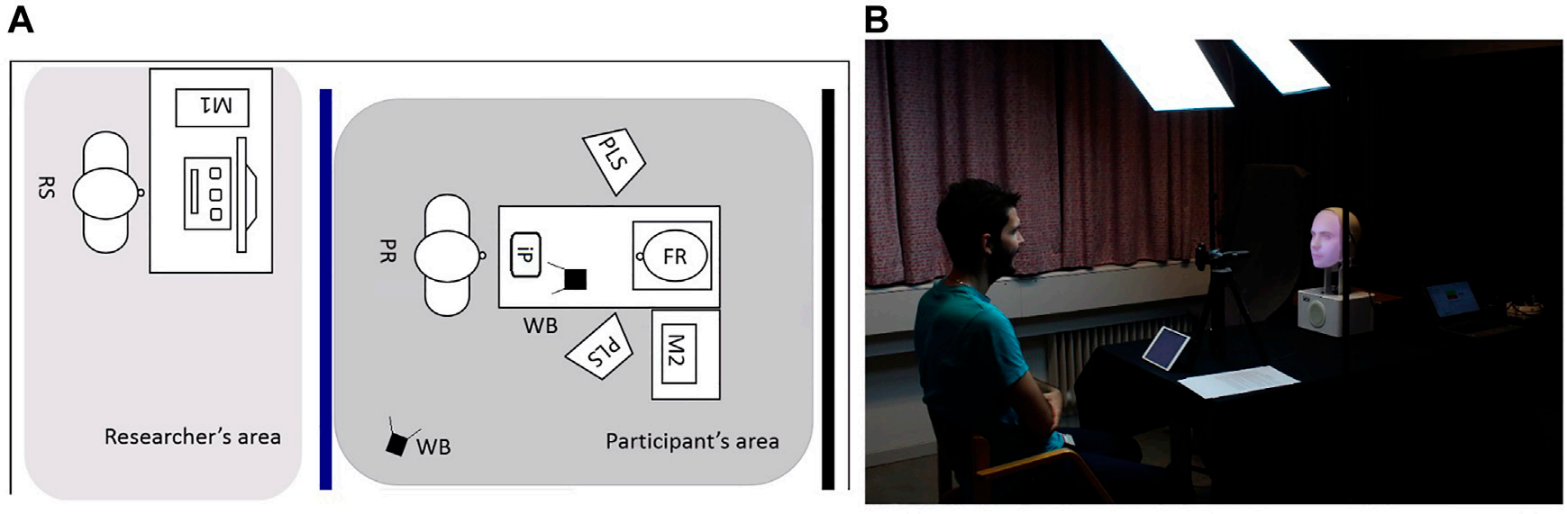
**(A)**: the experimental setup. **(B)**: A participant in the participant area.

The experimental sessions took place in a private laboratory room at Uppsala University (cf. Figure 4). To grant a feeling of privacy and an even background for the video-recordings, the participant’s area was separated from the researcher’s area by a blue curtain. Black curtains positioned behind the Furhat robot (FR) and the screen displaying the other agents ensured a good visibility of the agents from the participants’ perspective. Uniform lighting for the recordings was guaranteed through a professional lighting system (PLS) composed by two lamps. These were the only light sources in the experiment space. As both Furhat and the screen displaying the agents were sources of light themselves, the dark environment ensured a good visibility of the facial expressions.

The participant (PR) was sitting in the participants’ area at a distance of about 100 cm from the Furhat robot or the screen. This value falls in the *personal space* of the participants according to Hall (1969). The agents were thus close enough to the participants to be properly seen, but not too close to elicit an intimidating feeling. The agents were placed on a table at approximately 100 cm from the ground, which was roughly at eye level for the majority of participants. The video-recorded and the virtual agents were presented on a screen in portrait orientation. Their size was calibrated to match the size of Furhat’s head. All embodiments were controlled by a desktop computer (M1). An iPad (iP), placed on the table in front of the participant, was used for answering the questionnaires.

### 4.5 Measures

#### 4.5.1 Facial Recordings

To record participants’ faces, we used two LOGITECH C920HD PRO webcams (WB) with a 800 x 600 resolution, operating at 30 fps. The webcams were placed on top of a tripod. One was positioned in front of participants, at approximately 60 cm from them, and slightly on their side to not occlude the stimulus. The second was positioned on the side of the participant (cf. Figure 4). The webcams were connected to a laptop (M2) which was used to start, stop, and control the video-recordings during the experiment. Each webcam recorded the entire experimental session with the exclusion of the break between the spontaneous and instructed mimicry trials. Hence, we obtained two video files per camera, participant and session. The video-recordings of the frontal camera were used to assess participants’ mimicry, those of the lateral camera to capture the entire experimental scene.

#### 4.5.2 Questionnaires

Throughout the experiment, four different questionnaires were used. Questionnaire Q1 consisted of a general demographic questionnaire (10 items), the short version of the Big Five personality traits (10 items, Rammstedt and John, 2007) and the Interpersonal Reactivity Index (IRI, 21 questions, excluded personal distress, Cronbach’s *α* between 0.70 and 0.78 according to Davis et al. (1980). This questionnaire gauged the empathy and personality traits of the participants, and hence was not used to answer this paper’s research questions.

Questionnaire Q2 was shown to participants after every facial expression they observed to assess the emotion they recognized in the stimulus. It was composed of the question “Which of these facial expressions was just displayed?” with the six basic emotions, “neutral” and “I don’t know” as response options, and the question: “How certain are you of the selection you made in question 1?” with a three point Likert scale using the labels: “Uncertain”, “Neither nor”, “Certain”. The response options in the first question were displayed in one of three pre-shuffled orders to prevent a bias towards the first item on the scale.

Questionnaire (Q3) was shown after every embodiment in the first part of the experiment (i.e., spontaneous mimicry trial) to measure participants’ perceptions of the agents on four dimensions:

• *Anthropomorphism* (5 items, 5-point Likert scale), sub-scale from the Godspeed questionnaire by Bartneck et al. (2009) (Cronbach’s *α* = .91 according to (Ho and MacDorman, 2010).
• *Social presence* (8 items, 5-point Likert scale), excerpt from the social presence questionnaire developed by Harms and Biocca (2004). Sub-scales: co-presence (2 items, *α* = .84), Attentional Allocation (2 items, *α* = .81), Perceived Affective Understanding (2 items, *α* = .86), Perceived Emotional Interdependence (1 item, *α* = .85) and Perceived Behavioral Interdependence (1 item, *α* = .82).
• *Uncanniness and Likability* (10 items, 5-point Likert scale), excerpt from Rosenthal-von der Pütten and Krämer (2014), sub-scales likability and perceived threat (Cronbach’s *α* > = 0.82 for both sub-scales).

The order of questions and items remained the same across all embodiments.

At the end of the experimental session, the experimenter performed a semi-structured interview with the participant. The interview covered potential previous interactions with the Furhat robot, whether participants found aspects in the appearance of one of the characters particularly eerie, and if they had the impression that some of the facial expressions they observed were more difficult to trace back to a specific emotion. This interview was used to gather additional information about the experiment, and was not used to answer any research question present in this paper.

### 4.6 Procedure

After arriving to the lab, participants were informed about the experimental procedure, signed a consent form and answered Q1 on the iPad in front of them.

**FIGURE 5:**
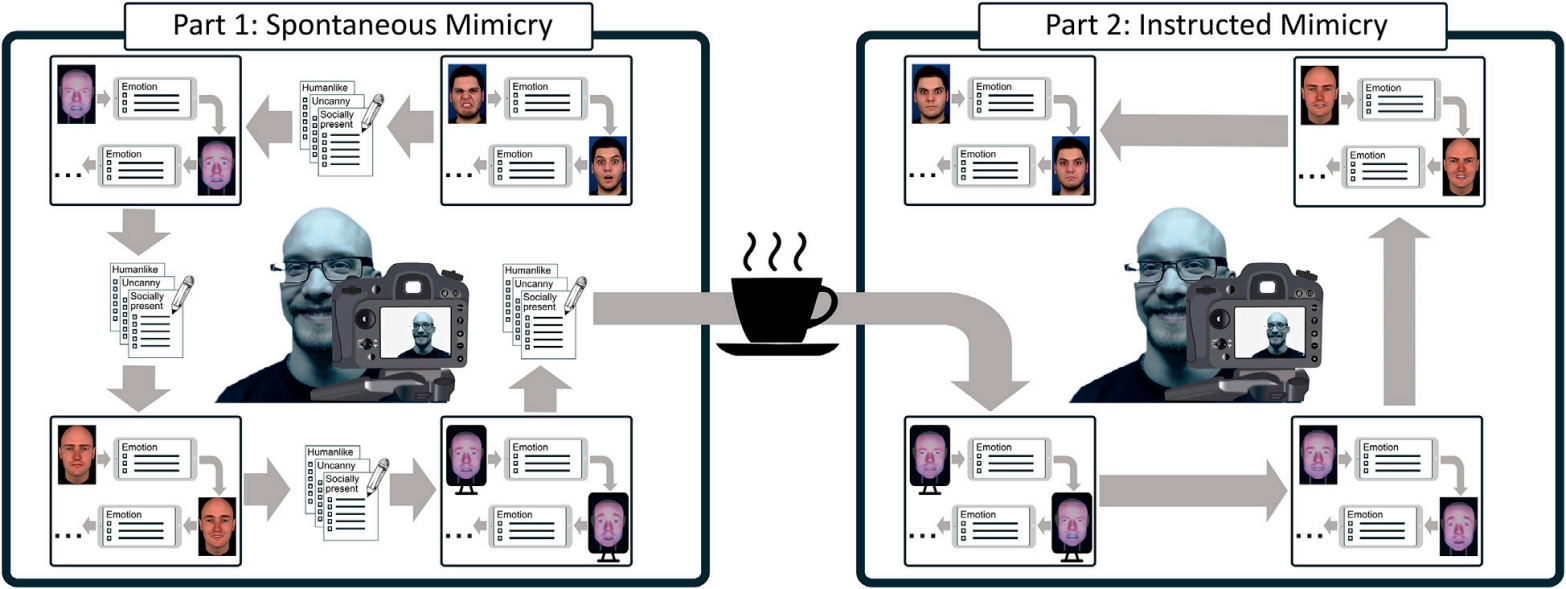
A visualization of the experiment procedure. Note that the order of embodiments as well as the facial expressions were shuffled between the experimental parts and participants to control for the ordering effect. In total, participants saw 12 expressions (2 x 6 emotions) per embodiment in each part.

For the first part of the experiment (cf. Figure 5A), participants were asked to first watch the facial expressions displayed by the four agents, which always started and ended with a beep tone, and then indicate which emotions they corresponded to using the questionnaire Q2 displayed on the iPad. Participants were also explained that, once they finished completing Q2 on the iPad and after a pause of about 2 s, the agent would automatically display the next facial expression preceded and followed by another beep tone, and the same procedure would be repeated until they had observed all facial expressions.

After participants observed all 12 expressions (2 trials x 6 emotions) for one embodiment, they rated their perception of the observed agent using questionnaire Q3 on the iPad. When necessary, the experimenter used this lapse of time to switch the physical robot with the screen. Once the participant finished responding to Q3, the stimuli for the subsequent embodiment were shown. Once participants responded to Q3 for the fourth and final agent of the spontaneous mimicry condition, they were given a 5 minute break and served refreshments.

For the second part of the experiment (cf. Figure 5B), participants were told that research suggests that mimicry increases emotion recognition. Therefore, they were asked to perform the same task once again, but this time by first mimicking the facial expression as accurately as possible and then noting down the emotion. The second part of the experiment followed the same procedure of the first part but the embodiments were re-shuffled in order. As Q3 was omitted for the second part of the experiment, participants had a shorter break between embodiments. Participants were shown each emotional expression twice in each phase of the study to make the estimation of their facial mimicry more robust. They were asked to recognize every emotion they were shown, so as to control for a potential learning effect due to multiple exposures.

At the end of the session, the experimenter conducted the short semi-structured interview. This was followed by a debriefing in which the researcher explained the true nature and objective of the experiment. Participants were informed again that they could request the deletion of their data at any point in time.

## 5 Mimicry Processing

The strategy to segment the videos differed between spontaneous and instructed facial mimicry. In the first case, we were interested in understanding whether people mimicked the observed facial expressions or not, whereas in the second case, we were interested in understanding how accurate people were in mimicking the dynamics of the observed facial expressions. This difference in focus is motivated by the different expected magnitudes of spontaneous and instructed facial mimicry. While the former is a subtle response that does not necessarily follow the same dynamics of the expression observed, the latter was expected to be a much stronger and accurate response due to its explicitly imitative nature.

For spontaneous facial mimicry, we annotated the frontal videos of the corresponding trial with the beginning and end of each stimulus in the ELAN 5.4. software. To do so, we used the audible beep tones that marked the start and end of each facial expression of the agents. We then used the minutes obtained from the annotation to automatically cut the original video into shorter snippets using ffmpeg^2^. To properly divide the instructed mimicry episodes, instead, we first manually identified the initial and final mimicry frames for each stimulus by closely examining the participant’s AU activation, and then we cut the original video a second before and after these frames. This process ideally led to 96 individual video snippets per participant, 48 for spontaneous and 48 for instructed facial mimicry.

Once the data were segmented, we deployed an automatic AU intensity detector to recognize which muscles of the participants’ face were activated in each video snippet of the spontaneous and instructed facial mimicry trials (cf. subsection 5.1). Then, in the case of spontaneous facial mimicry, we checked the AU time series to understand whether or not the target AU or combination of AUs amounting to each facial expression was active for a given lapse of time (cf. subsection 5.2). In the case of instructed facial mimicry, instead, we used the AU time series to perform a Cross-Recurrence Quantification Analysis (CRQA, Varni et al., 2017) as detailed in Section 5.3.

### 5.1 Detection of AU Activation

**FIGURE 6:**
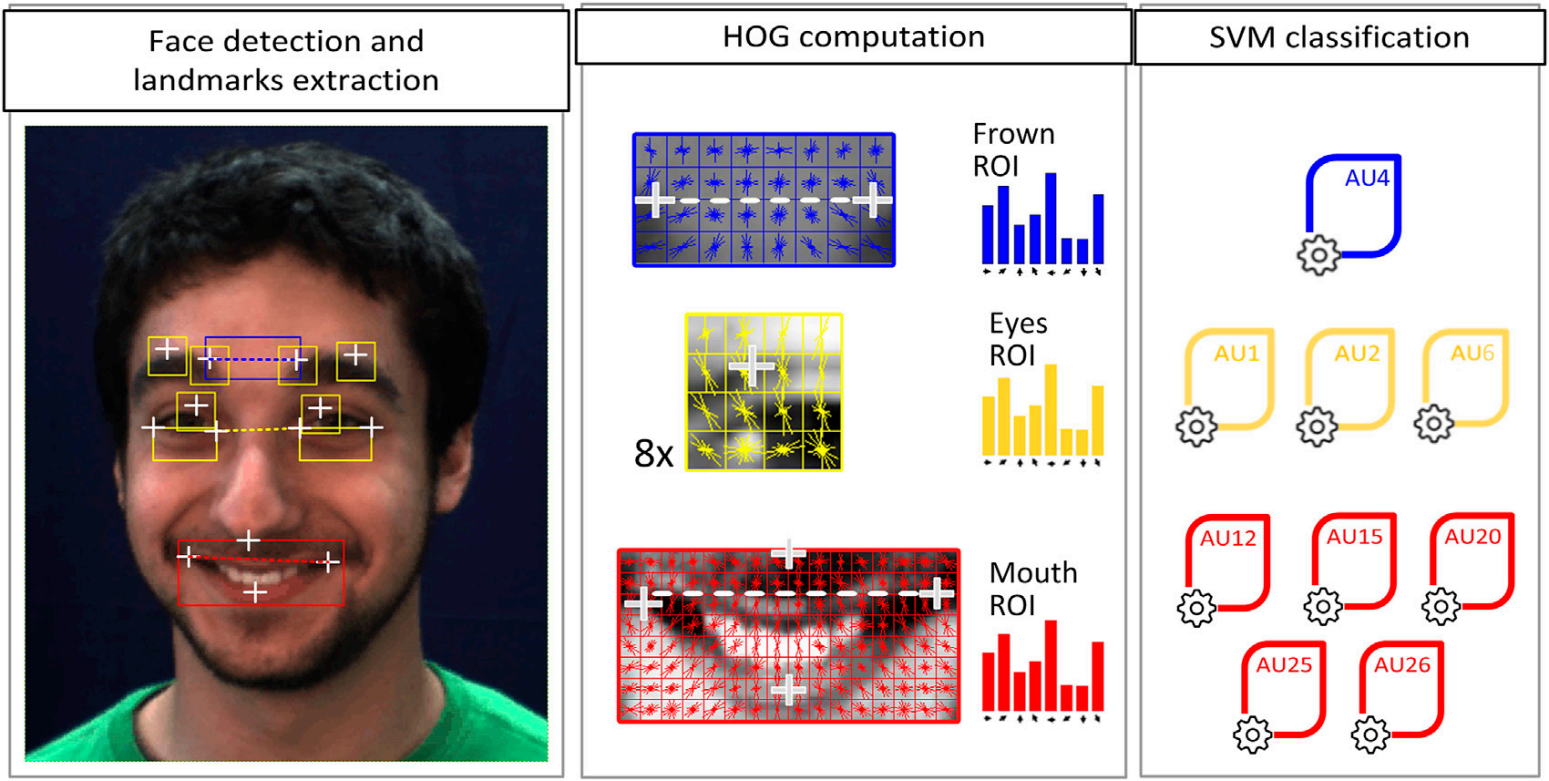
AU intensity detection pipeline. The white crosses represent the facial landmarks extracted from the face, while the dashed lines link the landmarks used for aligning each ROI.

The AU intensity detector used in this work is presented in Hupont and Chetouani (2019) and follows the pipeline shown in Figure 6. In a first step, it segments the face of the person from the whole input image and extracts a set of facial landmarks. Face segmentation is carried out by means of the (Viola and Jones, 2004’) Haar Cascade algorithm. The landmarks (14 white crosses in Figure 6) are extracted with the Intraface library introduced by Xiong and Torre (2013). On the basis of the facial landmark positions, three rectangular facial Regions of Interest (ROIs) are then defined and features of Histogram Oriented Gradients (HOG, Dalal and Triggs, 2005) are computed for each one of them. The ROIs used in our pipeline are:

• Frown ROI (used for AU4 model): This ROI is located around the inner eyebrow landmarks, which are also used for alignment purposes.
• Eyes ROI (AU1, AU2, and AU6): This ROI is made up of 8 patches located around the inner eyebrows, the middle eyebrows and the eye landmarks. ROI alignment is performed using inner eye corners. The final descriptor results from the concatenation of the 8 HOG descriptors.
• Mouth ROI (AU12, AU15, AU20, AU25, and AU26): This ROI is bounded by the nose center, the two lip corners and the lower lip. Alignment is done with respect to the lip corner positions.

Finally, the classification of each AU in terms of intensity is performed by an individually pre-trained Support Vector Machine (SVM) model using its corresponding ROI features as input. The SVM models were trained on the large-scale DISFA facial action database (Mavadati et al., 2013). Each model detects the activation of its corresponding AU in terms of six intensity categories, which are, according to Ekman’s taxonomy, (Hager et al., 2002): “N” (neutral), “A” (trace), “B” (slight), “C” (marked), “D” (severe), and “E” (maximum). The AU detector achieved an overall Intraclass Correlation Coefficient *ICC(3,1)* of 0.73, which is within state-of-the-art performances in the task of AU intensity detection.

The AU intensity time series was low-pass filtered through a centered moving average filter with a window size of 10 samples (33.3 ms). This filtering was applied to both the spontaneous and instructed facial mimicry time series. Moreover, the duration of time for which each AU was activated was also computed. For instructed facial mimicry, the first and the last 30 samples corresponding to the 1 s buffer left before and after the initial and final mimicry frames were removed in the final time series.

### 5.2 Processing of Spontaneous Facial Mimicry

To assess spontaneous facial mimicry, we divided the AU time series into two time intervals. The first time interval spanned from 0 to 1,000 ms after stimulus onset and encompassed quick mimicry responses occurring at a subperceptual level, which Dimberg et al. (2000) call Rapid Facial Reactions (RFR). The second time interval ranged from 1,000 to 5,000 ms after stimulus onset and comprised facial mimicry responses occurring at a more conscious level, which we call Controlled Facial Reactions (CFR).

To consider a facial expression as mimicked at each time interval (RFR, CFR), we checked whether the AU or combination of AUs corresponding to the target facial expression (cf. Table 1 based on Ekman et al., 2002) was active for at least 3 consecutive frames (100 ms). The activation was coded as 0 (not activated) or 1 (activated) and the intensity of the activation was not considered for this analysis as we expected the intensity of spontaneous facial mimicry to be low. We chose the threshold of 100 ms based on Ito et al. (2004), who defined this as the shortest period of time a muscle can take to move. To perform the statistical analyses, we calculated the percentage of spontaneous facial mimicry for RFR and CFR. This value was obtained *per embodiment* by dividing the number of trials in which the participant mimicked the facial expressions by the number of valid video snippets for that embodiment. Since in the spontaneous mimicry part of the study, participants were not explicitly asked to mimic the facial expressions they observed, in some snippets their faces were occluded, out of frame, or not recognizable by the AU intensity detector. These snippets were excluded from the final analyses. If more than half of the snippets of a particular embodiment were missing, we also excluded the other valid snippets from the a final analysis. Overall, this led to the exclusion of a total of 465 snippets for RFR (22%) and 394 for CFR (18%), and left us with 1,695 valid snippets for RFR, and 1766 for CFR.

**TABLE 1:** AUs or combination of AUs used to detect the spontaneous mimicry of the facial expressions of the six basic emotions (based on Ekman et al. (2002))

Emotion	Action units (AUs)
Anger	AU4
Disgust	AU4 + AU25
Fear	AU20, AU1 + AU2 + AU4
Happiness	AU6, AU12, AU6 + AU12
Sadness	AU1, AU15, AU1 + AU4
Surprise	AU26, AU1 + AU2

### 5.3 Analysis of Instructed Facial Mimicry

**FIGURE 7:**
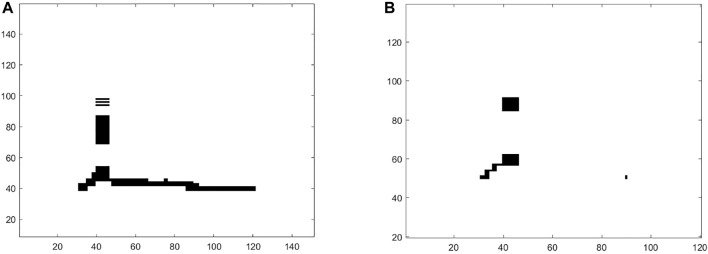
Cross-Recurrence Plot of two different participants intentionally mimicking the facial expressions of anger displayed by the virtual agent **(A)** and the physical Furhat **(B)**.

In order to accurately assess the dynamics of facial expressions, we performed a CRQA analysis (Marwan et al., 2007). CRQA is a technique enabling a quantitative measure of the graphical patterns occurring in a Cross-Recurrence Plot (CRP, cf. Figure 7). CRP is a plot looking at the times at which the features of a dynamical system *recur* (i.e., it is *close*) to features of another dynamical system. In this study, the two dynamical systems were the user and the artificial agents/video-recorded human, and the features were the AU intensities.

A CRP can be displayed as a square/rectangular black and white area spanned by two time series describing two systems. Black points correspond to the times at which the two systems co-visit the same area in the feature space, whereas white points correspond to the times at which each system runs in a different area. A CRP is expressed by the following *cross-recurrence matrix* (CR):

$$CR_{i,j}^{\vec{f1}\vec{f2}}\left(\epsilon \right)=\Theta \left(\epsilon -\|\vec{f1_{i}}-\vec{f2_{j}}\|\right),\;i=1\ldots N,j=1\ldots M$$ (1)

where $\vec{f1}$ and $\vec{f2}\;\in \;{I\;R}^{d}$ are the d-dimensional time series of the two systems having N and M samples, respectively; *ϵ* is the threshold to claim closeness between two points, Θ(.) is the Heaviside function and ‖.‖ is a norm. In this study, $\vec{f1}$ and $\vec{f2}\;\in \;{I\;R}^{3}$ are the time series of the AU intensities of the human and the artificial agents/video-recorded human over N samples. The threshold *ϵ* was set to 2 expressing that there was a match only when the ‘distance’ between the intensities of corresponding AUs was less than two. The norm used was the Manhattan distance.

CRPs can be analyzed through the Cross-Recurrence Quantification Analysis (CRQA) that enables to extract quantitative information from the black and white patterns appearing in the plot (see Marwan et al., 2007 for a complete survey). Typical patterns are: single isolated points, periodical diagonal lines, and vertical/horizontal lines. These patterns are hints of randomness, periodicity and laminar states of the dynamics of the system. In this study, we focused on the following CRQA measures (Marwan et al., 2007):

#### 5.3.1 Cross-Recurrence Rate (cRR)

The Cross-Recurrence Rate is defined as:

$$cRR\left(\epsilon \right)=\frac{1}{N^{2}}\overset{N}{\underset{i,j=1}{\sum }}CR_{i,j}\left(\epsilon \right)$$ (2)

and measures the density of recurrence points in a CRP. It corresponds to the ratio between the number of the matrix elements shared by the participant and the artificial agents/video-recorded human and the number of available elements (i.e. all the elements in the matrix). Here, *cRR* represents the overall extent to which the human and the artificial agent/recorded human were activating the same AUs at a similar level. This measure alone, however, even if it is a first measure to address mimicry, does not provide any information about how mimicry unfolds over time. To extract information about that, three other CRQA measures were computed:

#### 5.3.2 Average Diagonal Lines Length and Maximum Diagonal Line Length (*L*_max_)

*L* represents the average length of a recurrent trajectory in a CRP. It is defined as:

$$L=\frac{\sum_{l=l_{m}}^{N}lP\left(l\right)}{\sum_{l=l_{m}}^{N}P\left(l\right)}$$ (3)

where *l*_*m*_ is the minimal diagonal length to be taken into account, and *P*(*l*) is the histogram of the diagonal lines. The minimal diagonal length was set to 8 samples, i.e. around 250 ms (Fasel and Luettin, 2003). The value of L expresses how stable a recurrent trajectory is. Here high values of *L* correspond to long, almost identical portions of AU intensities of the human and the artificial agents over time. Moreover, the length *L*_max_ of the longest diagonal line in the CRP was computed. A large value of *L*_max_ shows a slow divergence of the AUs’ intensity trajectories.

#### 5.3.3 Determinism

As a fourth and last measure, the determinism was computed. It is defined as:

$$DET=\frac{\sum_{l=l_{m}}^{N}lP\left(l\right)}{\sum_{l=1}^{N}P\left(l\right)}$$ (4)

It measures the percentage of the cross-recurrence points forming diagonal lines (of at least length *l*_*m*_) computed with respect to all the cross-recurrence points in the CRP. *DET* ranges in [0, 1] and it is a hint of the predictability of the system (when *DET* = 0 the systems is stochastics, when *DET* = 1 it is periodic). In this study, high values of *DET* were expected to be found during good mimicry episodes.

While participants paid more attention to stay in frame during the instructed mimicry phase, we still had to exclude snippets due to occlusions and errors of the AU intensity detector. If there were only 5 or less valid snippets for a particular embodiment and participant, these were removed from the final analysis. Overall, we excluded a total of 209 snippets (9%) and were left with 1951 valid snippets for the analysis of instructed mimicry. For the statistical analysis, we calculated the average *cRR*, *L*_max_, *L*_*avg*_, and *DET* of each participant across all valid trials associated with one embodiment.

## 6 Results

In the remainder of the paper, we use: 1) *social presence* to refer to the dependent variables co-presence, attentional allocation, perceived affective understanding, perceived emotional interdependence, and perceived behavioral interdependence; 2) *perception of the agent* to refer to the dependent variables anthropomorphism, likability, and perceived threat; 3) *emotion recognition* to refer to the dependent variables percentage of correctly recognized emotions and average confidence in the recognized emotion; 4) *spontaneous facial mimicry* to refer to the percentage of spontaneous facial mimicry for rapid facial reactions (RFR) and controlled facial reactions (CFR); 5) *instructed facial mimicry* to refer to the average (avg) *cRR*, avg *L*, avg *L*_max_, and avg *DET*.

For the two manipulation checks (MC1 and MC2) and the preliminary analyses (PA), and for answering RQ1 and RQ2, we performed separate 3 x 3 mixed measures ANOVAs with humanlikeness as *between-subject factor* (i.e., humanlike, characterlike, morph), embodiment as *within-subject factor* (i.e., virtual agent, physical robot, and video-recording of the physical robot) and 1) social presence (MC1), 2) perception of the agent (MC2), 3) emotion recognition (PA), 4) spontaneous facial mimicry (RQ1) and 5) instructed facial mimicry (RQ2) as *dependent variables*. All *p*-values that we report in the post-hoc analyses are Bonferroni corrected to account for multiple tests.

For MC2, PA, RQ1, and RQ2, we also ran follow-up 2 x 3 mixed measures ANOVAs with humanlikeness as a between-subject factor (humanlike, characterlike, morph), *artificiality of the agent* as a within-subject factor (i.e., artificial agents and human agent), and the same dependent variables. To perform these analyses, we calculated the average value across all three artificial agents on each dependent variable. Social presence (MC1) was excluded from this set of analyses since the video-recorded human did not vary in embodiment like the artificial agents. We kept humanlikeness as a between-subject factor to control for eventual effects of the different levels of humanlikeness of the artificial agents on the dependent variables. However, as this effect is already covered by the 3 x 3 mixed measures ANOVAs, for the sake of brevity, we do not report these results. All the *p*-values that we report in the post-hoc analyses are Bonferroni corrected to account for multiple tests.

Finally, for answering RQ3 and RQ4, we performed separate regression analyses using spontaneous facial mimicry as a predictor of social presence and perceptions of the artificial agents (RQ3) and instructed facial mimicry as a predictor of spontaneous facial mimicry (RQ4). As RQ3 specifically focused on artificial agents’ facial mimicry, we used only the data from the artificial agents to perform the regression analyses. On the contrary, as RQ4 focused on facial mimicry in general and not specifically on artificial agents’ mimicry, we included also the data from the human video in the regression analyses.

### 6.1 Manipulation Check and Preliminary Analyses

#### 6.1.1 Manipulation Check: Social Presence of the Artificial Agents

The results indicated a significant main effect of embodiment on co-presence, affective understanding, and emotional interdependence (cf. Table 2 for the complete results). Furthermore, they showed a significant interaction effect of humanlikeness and embodiment on co-presence.

**TABLE 2:** Results of 3x3 Mixed Measures ANOVAs for Manipulation checks and RQ1 and RQ2. The significant results are displayed in bold, while the trend effects are presented in italics.

	Embodiment			Humanlikeness			Embod. x human		
**Social presence**	*F*(2, 80)	*p*	*ηp* ^2^	*F*(2, 40)	*p*	*ηp* ^2^	*F*(4, 80)	*p*	*ηp* ^2^
Co-presence	**7.878**	**0.001**	**0.165**	*2.719*	*0.078*	*0.120*	**4.036**	**0.005**	**0.168**
Att. Allocation	2.040	0.137	0.049	0.036	0.965	0.002	1.377	0.249	0.064
Aff. Understand	**3.643**	**0.031**	**0.083**	1.115	0.338	0.053	*2.373*	*0.059*	*0.106*
Em. Interdep	**5.864**	**0.004**	**0.128**	2.157	0.129	0.097	0.668	0.616	0.032
Beha. Interdep	0.630	0.535	0.015	0.750	0.479	0.036	0.725	0.578	0.035
**Agent’s Percept**	*F*(2, 80)	*p*	*ηp* ^2^	*F*(2, 40)	*p*	*ηp* ^2^	*F*(4, 80)	*p*	*ηp* ^2^
Anthropomorph	**15.587**	<.001	**0.280**	**3.399**	**0.043**	**0.145**	*2.246*	*0.071*	*0.101*
Perceived Threat	**6.470**	**0.002**	**0.139**	0.244	0.785	0.012	*2.447*	*0.053*	*0.109*
Likability	**8.361**	**0.001**	**0.173**	1.776	0.182	0.082	1.454	0.224	0.068
**Emo. Recogn**	*F*(2, 80)	*p*	*ηp* ^2^	*F*(2, 40)	*p*	*ηp* ^2^	*F*(4, 80)	*p*	*ηp* ^2^
Recogn. (Spont.)	0.296	0.745	0.007	**4.004**	**0.026**	**0.167**	0.253	0.907	0.013
Recogn. (Instr.)	1.147	0.323	0.028	**5.540**	**0.008**	**0.217**	0.482	0.749	0.024
**Spont. Mimicry**	*F*(2, 64)	*p*	*ηp* ^2^	*F*(2, 32)	*p*	*ηp* ^2^	*F*(4, 64)	*p*	*ηp* ^2^
Freq. RFR	**9.336**	<.001	**0.226**	1.002	0.378	0.059	1.071	0.378	0.063
Freq. CFR	**4.645**	**0.013**	**0.127**	0.636	0.536	0.038	1.566	0.194	0.089
**Instr. Mimicry**	*F*(2, 76)	*p*	*ηp* ^2^	*F*(2, 38)	*p*	*ηp* ^2^	*F*(4, 76)	*p*	*ηp* ^2^
Avg *cRR*	0.097	0.908	0.003	2.189	0.126	0.103	0.785	0.538	0.040
Avg *L*	0.364	0.696	0.009	0.208	0.813	0.011	0.411	0.800	0.021
Avg *L*_max_	0.477	0.662	0.012	0.293	0.748	0.015	0.298	0.878	0.015
Avg *DET*	0.219	0.804	0.006	0.187	0.830	0.010	0.784	0.539	0.040

Post-hoc analyses uncovered that the virtual agent was perceived as significantly more co-present than the video-recorded robot (*p* = .005, cf. Table 3 for the descriptive statistics), and the physical robot was perceived as significantly more co-present (*p* = .005) than the video-recorded one. No such difference was observed between the virtual agent and the physical robot (*p* = 1.00). Moreover, they disclosed that participants perceived their affective understanding of the physical robot to be significantly higher than that of the virtual agent (*p* = .045), while the virtual agent and the video-recorded robot did not differ in terms of perceived affective understanding (*p* = .255), and neither did the physical robot and the video-recorded one (*p* = 1.00). Finally, participants perceived significantly higher emotional interdependence with the physical robot with respect to both the virtual agent (*p* = .021, cf. Table 3 for the descriptive statistics) and the video-recorded robot (*p* = .019). No such difference was present between the virtual agent and the video-recorded robot (*p* = 1.00).

**TABLE 3:** Descriptive Statistics of the 3 x 3 Mixed Measures ANOVAs per Embodiment: Mean (*M*) and standard deviation (*SD*) of all dependent variables.

	Virtual agent		Physical robot		Video robot	
	*M*	*SD*	*M*	*SD*	*M*	*SD*
Co-presence	3.87	0.832	3.95	0.844	3.59	0.847
Att. Allocation	4.05	0.837	4.20	0.757	4.02	0.809
Aff. Understanding	3.17	0.778	3.45	0.625	3.27	0.658
Em. Interdependence	1.79	0.888	2.12	1.051	1.70	0.832
Beha. Interdependence	2.28	1.076	2.35	1.066	2.21	0.914
Anthropomorphism	2.53	0.834	3.10	0.742	2.67	0.777
Perceived Threat	2.10	0.769	1.84	0.650	1.72	0.524
Likability	2.22	0.759	2.66	0.708	2.43	0.787
Recognized (Spont.)	0.77	0.139	0.79	0.139	0.77	0.129
Recognized (Instr.)	0.80	0.151	0.77	0.142	0.79	0.117
Freq. RFR	0.56	0.165	0.53	0.189	0.66	0.151
Freq. CFR	0.74	0.174	0.66	0.188	0.73	0.156
Avg *cRR*	13.12	7.430	13.54	6.692	13.26	6.585
Avg *L*	5.52	2.987	5.29	2.754	5.66	1.656
Avg *L*_max_	6.59	3.596	6.36	3.364	6.88	3.321
Avg *DET*	2.78	1.856	2.63	1.612	2.78	1.262

Further follow-up post-hoc analyses on the interaction effect of humanlikeness and embodiment on co-presence uncovered that, in the characterlike condition, the virtual agent (*M* = 4.10, SD  = .632) and the physical robot (*M* = 4.27, SD  = .729) were perceived as significantly more co-present than the video-recorded robot (*M* = 3.70, SD  = .621, virtual agent: *p* = .026; physical robot: *p* = .028), but they did not significantly differ in co-presence from each other (*p* = 1.00). Likewise, in the morph condition, the virtual agent (*M* = 3.71, SD  = 1.051) was perceived as significantly more co-present than the video-recorded robot (*M* = 3.07, SD  = .938, *p* = .016), the physical robot (*M* = 3.53, SD  = .930) was perceived as significantly more co-present than the video-recorded one (*p* = .051), and the virtual agent and the physical robot did not differ from each other (*p* = .409). Interestingly though, in the humanlike condition (virtual agent: *M* = 3.79, SD  = .777; physical robot: *M* = 4.04, SD  = .746; video-recorded robot: *M* = 4.00, SD  = .734), these differences between artificial agents were not present (virtual agent - physical robot: *p* = .331; virtual agent–video-recorded robot: *p* = .083; physical robot - video-recorded robot: *p* = 1.00).

#### 6.1.2 Manipulation Check: Perception of the Agents

When checking for differences in the perception of the artificial agents across levels of humanlikeness and embodiments, we found a significant main effect of embodiment on anthropomorphism, perceived threat, and likability (cf. Table 2 for the complete results) and a significant main effect of humanlikeness on anthropomorphism.

Bonferroni-corrected post-hoc analyses revealed that the virtual agent and the video-recorded robot were perceived as less anthropomorphic than the physical robot (both *p* < .001, cf. Table 3 for the descriptive statistics), but the virtual agent and the video-recorded robot did not differ in terms of anthropomorphism between each other (*p* < .682). Similarly, in terms of likability, the virtual agent (*p* = .001, cf. Table 3 for the descriptive statistics) and the video-recorded robot (*p* = .042) were perceived as less likable than the physical robot. However, the virtual agent and the video-recorded robot did not differ from each other (*p* = .291). Finally, concerning perceived threat, the virtual agent was perceived as more threatening than the video-recorded robot (*p* = .001, cf. Table 3 for the descriptive statistics), but no such difference was present between the virtual agent and the physical robot (*p* = .104) and between the video-recorded and the physical robot (*p* = .822).

With regards to the main effect of humanlikeness, the post-hoc analyses disclosed that humalike artificial agents were perceived as significantly more anthropomorphic than morph artificial agents (*p* = .046, cf. Table 4 for the descriptive statistics). However, humanlike and characterlike artificial agents (*p* = .232) and characterlike and morph agents (*p* = 1.00) did not differ significantly from each other.

**TABLE 4:** Descriptive Statistics of the 3x3 Mixed Measures ANOVAs per level of humanlikeness: Mean (*M*) and standard deviation (*SD*) of all dependent variables.

	Character		Humanlike		Morph	
	*M*	*SD*	*M*	*SD*	*M*	*SD*
Co-presence	4.02	0.720	3.94	0.722	3.44	0.722
Att. Allocation	4.10	0.734	4.12	0.737	4.05	0.737
Aff. Understanding	3.19	0.550	3.48	0.550	3.24	0.550
Em. Interdependence	2.20	0.775	1.64	0.775	1.74	0.775
Beha. Interdependence	2.16	0.910	2.52	0.909	2.17	0.909
Recognized (Spont.)	0.83	0.089	0.74	0.090	0.76	0.090
Recognized (Instr.)	0.85	0.108	0.72	0.109	0.79	0.109
Anthropomorphism	2.69	0.631	3.11	0.632	2.51	0.632
Perceived Threat	1.87	0.500	1.96	0.501	1.83	0.501
Likability	2.40	0.620	2.67	0.621	2.23	0.621
Freq. RFR	0.56	0.137	0.54	0.135	0.62	0.137
Freq. CFR	0.68	0.144	0.70	0.144	0.75	0.144
Avg *cRR*	15.75	5.863	11.07	5.863	12.95	5.863
Avg *L*	5.53	2.312	5.17	2.311	5.74	2.312
Avg *L*_max_	6.82	2.862	6.11	2.859	6.87	2.862
Avg *DET*	2.60	1.310	2.91	1.309	2.70	1.310

When running the 2 x 3 ANOVA focusing on the agents’ artificiality, we found out that the video-recorded human was perceived as significantly more anthropomorphic (*p* < .001), more likable (*p* < .001), and less threatening (*p* < .001) than the artificial agents (cf. Table 5 for the results and the descriptive statistics).

**TABLE 5:** Results of 2 x 3 ANOVAs and Descriptive Statistics. The significant results are displayed in bold. The Mean (*M*) and standard deviation (*SD*) of all dependent variables are divided per Artificial and Human Agents.

	Artificiality			Artif. Agents		Human video	
**Agent’s percept**	*F*(1, 41)	*p*	*ηp* ^2^	*M*	*SD*	*M*	*SD*
Anthropomorphism	**130.064**	<.001	**0.760**	2.77	0.659	4.13	0.761
Perceived Threat	**29.800**	<.001	**0.421**	1.93	0.544	1.51	0.451
Likability	**39.159**	<.001	**0.489**	2.43	0.626	3.00	0.757
**Emo. Recogn**	*F*(2, 40)	*p*	*ηp* ^2^	*M*	*SD*	*M*	*SD*
Recognized (Spont.)	1.470	0.233	0.035	0.78	0.096	0.75	0.151
Recognized (Instr.)	**7.494**	**0.009**	**0.158**	0.79	0.119	0.84	0.120
**Spont. Mimicry**	*F*(1, 32)	*p*	*ηp* ^2^	*M*	*SD*	*M*	*SD*
Freq. RFR	**34.835**	<.001	**0.521**	0.60	0.146	0.84	0.127
Freq. CFR	**4.323**	**0.046**	**0.119**	0.74	0.144	0.79	0.156
**Instr. Mimicry**	*F*(1, 39)	*p*	*ηp* ^2^	*M*	*SD*	*M*	*SD*
Avg *cRR*	0.653	0.424	0.016	13.92	7.172	13.29	10.124
Avg *L*	0.039	0.844	0.001	5.56	2.288	5.53	2.697
Avg *L*_max_	0.206	0.652	0.005	6.70	2.824	6.55	3.369
Avg *DET*	0.249	0.621	0.006	2.75	1.270	2.65	1.638

##### 6.1.2.1 Discussion of Manipulation Check

As specified in Section 2.4, the artificial agents and the video-recorded human differed as follows: 1) the physical robot was *artificial, physically embodied*, and *co-present*; 2) the virtual agent was *artificial, virtually embodied*, and *co-present*; 3) the video-recorded robot was *artificial, physically embodied*, but *not co-present*; and 4) the video-recorded human was *natural, physically embodied*, but *not co-present*. The manipulation checks that we performed were aligned with these differences. Indeed, the video-recorded robot was perceived as significantly less co-present than the virtual agent and physical robot. Furthermore, the artificial agent that was physically embodied and co-present (i.e., the physical robot) was perceived as easier to understand affectively, more anthropomorphic, more likable, and elicited more emotional understanding than the other artificial agents. Finally, the human agent was perceived as more anthropomorphic, more likable, and less threatening than the artificial agents. As a result, we can state that the manipulation of embodiment worked as expected in this study.

With regards to the manipulation of humanlikeness, the core dependent variable that we expected to change was anthropomorphism. The characterlike and morph robot did not differ in anthropomorphism and neither did the characterlike and humanlike robot. However, in line with our expectations, the humanlike robot was perceived as more anthropomorphic than the morph robot. As a result, we considered the manipulation of humanlikeness only partially successful. This is surprising given that the characterlike version has a clearly sketched appearance compared to the humanlike version, which is derived from a human picture. It is interesting to note, however, that all three versions had a comparable high rating of humanlikeness, which could potentially be explained by the very humanlike appearance of the Furhat robot platform itself. Both the humanlike and the characterlike facial texture may have hence elicited a congruent and overall humanlike perception. The morph, on the contrary, may have received the lowest rating of humanlikeness due to the incongruence of the facial features. Even though this did not lead to an uncanny feeling in participants, it could have still decreased its anthropomorphism.

With regards to the manipulation of humanlikeness, it was also very interesting to discover that, when the appearance of the artificial agents was humanlike, the differences in co-presence between the different embodiments ceased to exist. This result seems to suggest that the humanlike appearance has in itself a quality of co-presence that goes beyond the physical instantiation of an artificial agent.

### 6.2 Preliminary Analyses: Emotion Recognition

**FIGURE 8:**
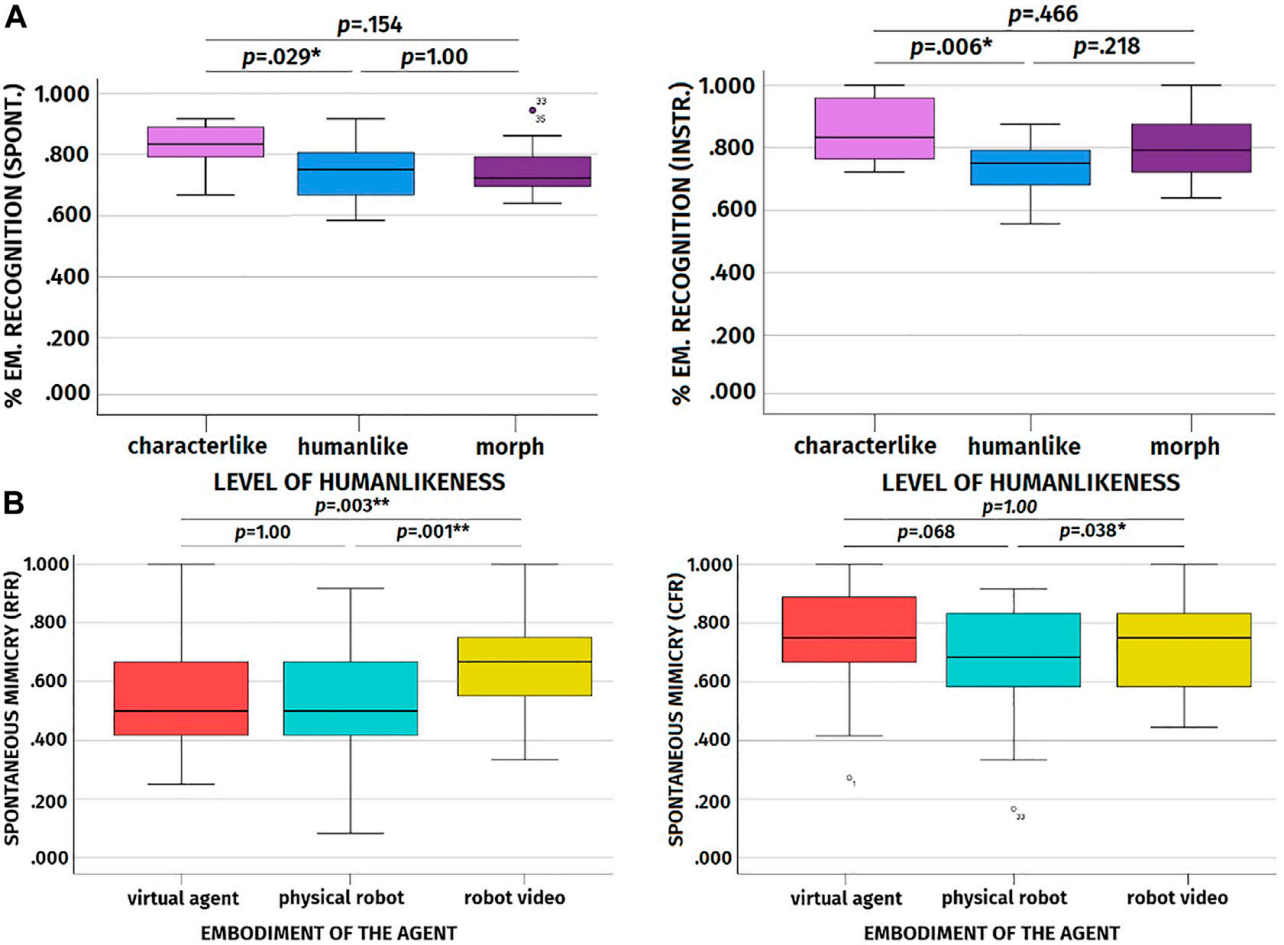
**(A)**: Boxplots showing the effect of level of humanlikeness on the percentage of emotions correctly recognized for the spontaneous and instructed mimicry trials. **(B)**: Boxplots showing the effect of the agent’s embodiment on frequency of spontaneous facial mimicry for RFR and CFR.

As a preliminary analysis, we checked whether participants’ ability to recognize the emotions displayed by the artificial agents differed across embodiments and levels of humanlikeness. Interestingly, we discovered a main effect of humanlikess on the percentage of emotion recognized (cf. Table 2 for the complete results). According to the results, participants’ emotion recognition was better when participants observed the characterlike agents with respect to when they observed the humanlike agents (cf. Table 4 for the descriptive statistics). This was true both in the spontaneous mimicry (*p* = .029) and in the instructed mimicry trials (*p* = .006, cf. Figure 8A). No such differences in emotion recognition were observed between characterlike and morph agents (spontaneous mimicry trial: *p* = .154; instructed mimicry trial: *p* = .466) and between morph and humanlike agents across trials (spontaneous mimicry trial: *p* = 1.00; instructed mimicry trial: *p* = .218). When it comes to the 2 x 3 ANOVAs focusing on the agents’ artificiality, we found a significant difference between artificial agents and the video-recorded human in terms of emotion recognition only for the instructed mimicry trial (cf. Table 5 for the results and descriptive statistics). In this case, the percentage of emotions correctly recognized was higher for the human with respect to the artificial agents.

#### 6.2.1 Discussion of Preliminary Analyses

As predicted, the facial expressions of the video-recorded human were easier to recognize in comparison to the facial expressions of the artificial agents. However, somewhat unexpectedly, and partially in conflict with this result, the facial expressions of the characterlike artificial agents were easier to recognize with respect to those of the humanlike artificial agents both for the spontaneous and instructed mimicry trials. We ascribe this results to the stylized appearance of the characterlike agents, which might have made their expressions more readable and recognizable than those of the other agents.

### 6.3 Results for Research Questions

#### 6.3.1 Influence of Embodiment and Humanlikeness on Spontaneous Facial Mimicry [RQ1]

Results disclosed a significant main effect of embodiment on spontaneous facial mimicry both for RFR and CFR (cf. Table 2 for the complete results). However, we did not find any significant effect of humanlikeness and embodiment and humanlikeness alone on spontaneous facial mimicry.

Post-hoc analyses with a Bonferroni correction disclosed that for the RFR the video-recorded robot was mimicked significantly more than the virtual agent (*p* = .003, cf. Table 3 for the descriptive statistics and cf. Figure 8B for the boxplot) and physical robot (*p* = .001), and that the physical robot and the virtual agent did not differ in spontaneous facial mimicry from each other (*p* = 1.00). With regards to CFR, the post-hoc analyses showed that the physical robot was mimicked significantly less than the video-recorded robot (*p* = .038, cf. Figure 8B for the boxplot), while the virtual agent and the video-recorded robot did not differ in terms of spontaneous facial mimicry (*p* = 1.00) and only a trend difference was present between the virtual agent and the physical robot (*p* = .068).

When taking into account the artificiality of the agent as the within-subject factor by analyzing the average mimicry frequency across all artificial agents in comparison to the mimicry frequency when observing the video recording of the human, we found a significant main effect of artificiality on spontaneous facial mimicry (cf. Table 5 for the results and the descriptive statistics). In this case, the video-recorded human was spontaneously mimicked significantly more than the artificial agent both for RFR and CFR.

##### 6.3.1.1 Discussion of RQ1

These results are somewhat complimentary to those we found for the manipulation checks. Indeed, it seems that the agent that elicited the highest ratings of co-presence, affective understanding, emotional interdependence, anthropomorphism, and likability, namely the physical robot, was also the agent that was spontaneously mimicked the least. If we take the facial mimicry-rapport hypothesis into account, this result is somewhat counterintuitive. Indeed, in line with this hypothesis, the robot eliciting the most favorable relational ratings should have been the one spontaneously mimicked the most. However, if we take the emotion recognition task into account, we can partially explain this result. Recognizing the emotions of another agent is an activity that implies putting some distance between the agent we observe and ourselves. It somewhat entails considering the agent we observe as a stimulus, rather than a relational agent. In this sense, we can hypothesize that an agent that is perceived as more socially present and elicits more positive perceptions is less good as a stimulus, it is more likely to act as a distractor, and hence can hinder the goal of the emotion recognition task. It is interesting to note that, when the agent is a human, this dynamic does not take place and the human is, as foreseeable, spontaneously mimicked more than the artificial agents. We can ascribe this result to the familiarity of the human stimulus. Indeed, the video-recorded human is undoubtedly more positively evaluated than the artificial agents and hence more likely to act as a distractor. However, it is also the stimulus with which participants are the most familiar and whose facial expressions they are more used to recognize.

#### 6.3.2 Influence of Embodiment and Humanlikeness on Instructed Facial Mimicry [RQ2]

The results of the 3 x 3 mixed measures ANOVAs did not show any significant effect of embodiment and humanlikeness on instructed facial mimicry (cf. Table 2 for the complete results). Similarly, the results of the 2 x 3 mixed measures ANOVA did not disclose any significant effect of the agents’ artificiality on participants’ instructed facial mimicry (cf. Table 5 for the results and the descriptive statistics).

##### 6.3.2.1 Discussion of RQ2

As opposed to Hofree et al. (2014), in our study, the results of instructed facial mimicry are not congruent with those of spontaneous facial mimicry. Based on our findings, we can state that *when facial mimicry is explicitly prompted, the agent’s appearance and embodiment cease to have an influence on it*. This might be due to the fact that, when facial mimicry transforms itself into a purely imitative act, it loses its social value, and hence those variables that would have likely affected it due to their relational value, such as the agents’ level of humanlikeness and their embodiment, do not influence it anymore. This assumption is further reinforced by the fact that people’s ability to mimic an agent as closely as possible does not differ also when artificial and human agents are taken into account.

#### 6.3.3 Spontaneous Facial Mimicry as Predictor of Perceived Social Presence and Perceptions of Artificial Agents [RQ3]

The results of the regression analyses in Table 6 show that spontaneous facial mimicry for RFR was a negative predictor of co-presence, attentional allocation, and affective understanding, whereas spontaneous facial mimicry for CFR was a negative predictor of attentional allocation and emotional interdependence. Moreover, they showed that spontaneous facial mimicry for RFR was a negative predictor of people’s perceptions of the artificial agents’ likability and anthropomorphism, and spontaneous facial mimicry for CFR was a negative predictor of participants’ perception of the agents’ likability (cf. Table 6 for the complete results).

**TABLE 6:** Regression Analyses [RQ3]. Frequency of spontaneous facial mimicry as predictor of Social Presence and Perceptions of Artificial agents. The significant results are displayed in bold, while the trend effects are presented in italics.

	Spont. Mimicry (% RFR)				Spont. Mimicry (% CFR)			
Dependent variables	*β*	*t*(149)	*p*	*r* ^2^	*β*	*t*(154)	*p*	*r* ^2^
Co-presence	**−0.183**	**−2.271**	**0.025**	**0.034**	**−**0.100	**−**1.246	0.215	0.010
Att. Allocation	**−0.211**	**−2.626**	**0.010**	**0.045**	**−0.230**	**−2.920**	**0.004**	**0.053**
Aff. Understanding	**−0.203**	**−2.527**	**0.013**	**0.041**	**−**0.127	**−**1.587	0.115	0.016
Em. Interdependence	**−**0.077	**−**0.940	0.349	0.006	**−0.204**	**2.581**	**0.011**	**0.042**
Beha. Interdependence	**−**0.076	**−**0.932	0.353	0.006	**−**0.098	**−**1.224	0.223	010
Anthropomorphism	**−0.168**	**−2.074**	**0.040**	**0.028**	**−**0.112	**−**1.388	0.167	0.012
Perceived Threat	0.055	0.666	0.506	0.003	0.124	1.546	0.124	0.015
Likability	**−0.191**	**−2.373**	**0.019**	**0.037**	**−0.262**	**−3.359**	**0.001**	**0.069**
% Recognized	**−** *0.169*	**−** *1.821*	*0.071*	*0.029*	**−**0.073	**−**0.783	0.435	0.005
Confidence Recogn	**−0.231**	**−2.520**	**0.013**	**0.053**	**−**0.121	**−**1.310	0.193	0.015

##### 6.3.3.1 Discussion of RQ3

These results are in line with those of RQ1 and seem to suggest that, in this study, the more the artificial agents were spontaneously mimicked, the less positive perceptions they elicited, the less socially co-present they were perceived, and the less people felt emotionally connected with them and capable of understanding their affective states. We assume that this result, which goes against most of the literature focusing on the social function of spontaneous facial mimicry, can be ascribed to the emotion recognition task in which participants were involved. Our hypothesis is that, within an emotion recognition task, spontaneous facial mimicry does not fulfill anymore a social function, but rather serves the purpose of emotion recognition. In this context, the embodiment people rated as the most anthropomorphic, likable and co-present (i.e., the physically present robot) was the one that people could relate to the most. Consequently, they might have had an easier time understanding its facial expressions, and hence less necessity to spontaneously mimic them.

To verify this assumption, we performed a few additional regression analyses using spontaneous facial mimicry for RFR and CFR as predictors and the percentage of correctly recognized facial expressions and the confidence in the recognized emotion as dependent variables. As we supposed, participants’ spontaneous facial mimicry was a significant negative predictor of their certainty of the correctness of the recognized emotion and a trend negative predictor of their emotion recognition performance (cf. Table 6 for the complete results). This indicates that *the more participants spontaneously mimicked the artificial agents, the less they were confident in the emotion they recognized*. Such a result is particularly important as it corroborates the theory that facial mimicry serves the purpose of emotion recognition, but only when the emotions to recognize are ambiguous (Hess and Blairy, 2001; Fischer et al., 2012).

#### 6.3.4 Instructed Facial Mimicry as Predictor of Spontaneous Facial Mimicry [RQ4]

The results of the regression analyses displayed in Table 7 show that the average *cRR*, *L*, *L*_max_, and *DET* are all significant negative predictors of spontaneous facial mimicry for RFR but they do not equally predict spontaneous facial mimicry for CFR.

**TABLE 7:** Regression Analyses [RQ4]. Features of instructed facial mimicry as predictors of the frequency of Spontaneous facial mimicry. The significant results are displayed in bold.

	Spont. Mimicry (% RFR)				Spont. Mimicry (% CFR)			
Predictors	*β*	*t*(141)	*p*	*r* ^2^	*β*	*t*(146)	*p*	*r* ^2^
Avg *cRR*	**−0.229**	**−2.783**	**0.006**	**0.052**	**−**0.093	**−**1.124	0.263	0.009
Avg *L*	**−0.186**	**−2.241**	**0.027**	**0.035**	**−**0.068	**−**0.819	0.414	0.005
Avg *L*_max_	**−0.175**	**−2.107**	**0.037**	**0.031**	**−**0.057	**−**0.683	0.496	0.003
Avg *DET*	**−0.212**	**−2.567**	**0.011**	**0.045**	**−**0.098	**−**1.189	0.236	0.010

##### 6.3.4.1 Discussion of RQ4

This result is extremely interesting as it suggests that, in this study, the more closely participants mimicked the facial expressions of the agents when instructed to do so, the less likely they were to spontaneously mimic the agents at an unconscious level of processing. Since we have seen that spontaneous facial mimicry for RFR was a negative predictor of participants’ confidence in the recognized emotion (and partially also of their ability to recognize the target emotion), it does not surprise that people that mimic an emotion well under instruction, actually mimic it less at a subperceptual level. Indeed, if people are better able to mimic all the temporal dynamics of a target facial expression, they might also be more capable of recognizing that target emotion. In this sense, as opposed to spontaneous facial mimicry, instructed facial mimicry might signal a better understanding of the emotion. This finding entails that, even though in an emotion recognition task, instructed facial mimicry does not behave similarly to spontaneous facial mimicry, it still maintains a relation with it.

## 7 General Discussion

This study investigated how the humanlikeness and embodiment of an artificial agent could influence people’s mimicry of its facial expressions. Based on Hofree et al. (2014), we expected that physically embodied, co-present, and humanlike artificial agents could elicit higher spontaneous and instructed facial mimicry than virtually embodied, non-co-present, and less humanlike ones, and that instructed facial mimicry could positively predict spontaneous facial mimicry. Moreover, based on the link between facial mimicry and rapport, we postulated that spontaneous facial mimicry could positively predict participants’ evaluations of the agents’ anthropomorphism, social presence, and likability, and negatively predict their perceived uncanniness. Although our manipulation of embodiment was successful and the one of humanlikeness partially successful, and the task we chose was taken from the existing literature (Hofree et al., 2014 and Kulesza et al., 2015, the results we obtained did not meet our expectations (cf. H1–H4 in Section 3). We found that: 1) the physically embodied, co-present artificial agent (i.e., the physical robot) was the one that was spontaneously mimicked the least regardless of its humanlikeness (cf. H1); 2) instructed facial mimicry did not behave congruently to spontaneous facial mimicry (cf. H2); 3) spontaneous facial mimicry negatively predicted anthropomorphism, social presence, and likability, and did not predict uncanniness (cf. H3); and 4) instructed facial mimicry negatively predicted spontaneous facial mimicry (cf. H4).

While these results were surprising, their consistency led to a hypothesis that some element of the task that was given to the participants hindered the social value of facial mimicry. Following the *automatic embodiment account* (Niedenthal et al., 2010), we postulated that the task’s focus on emotion recognition could have caused a change in the meaning of facial mimicry. Additional analyses confirmed our suspicion. In fact, they indicated that the spontaneous facial mimicry of the artificial agents was a significant negative predictor of participants’ confidence in the emotion recognized. This result seems to suggest that, *in the context of human-agent and human-robot mimicry, the emotion recognition goal of a task can flip the social value of spontaneous facial mimicry, and transform a physically embodied, co-present artificial agent into a distractor*. This may have arisen by chance due to elements of the study design and deserves further exploration and replication. Due to the only partially successful manipulation of the robot’s perceived humanlikeness, it also requires further investigation whether this effect is really independent of the robot’s level of anthropomorphism. The primary objective of this study was to understand whether spontaneous facial mimicry could be used as a cue of liking and rapport in HAI and HRI, and whether instructed facial mimicry could act as a proxy of spontaneous facial mimicry. Although our findings do not meet our expectations, the fact that they went in the exact opposite direction to our original hypotheses may suggest that, *in an emotion recognition task, spontaneous facial mimicry can still be used as a predictor of liking and rapport, and instructed facial mimicry could still function as a predictor of spontaneous facial mimicry, but they need to be envisioned as negative predictors rather than positive ones*. Additional work is needed to corroborate these preliminary results, and understand whether context alone (emotion recognition task vs social interaction) can influence the value of facial mimicry in HAI and HRI in the way we have described.

## 8 Limitations and Future Work

One limitation of the current experimental design is the focus on one particular robotic embodiment (i.e., the Furhat robot). While this platform has several advantages, like the easy alteration of facial features and expressions, it is sometimes difficult to discern facial detail clearly. By keeping the robot platform consistent across conditions, we could limit the influence of confounding factors on our results. However, this in turn reduced the strength of the manipulation of humanlikeness and could be the reason why we did not see the agents’ anthropomorphism differ between the characterlike and humanlike, and the characterlike and morph conditions. Future studies should hence investigate facial mimicry in emotion recognition tasks carried out with multiple humanoid robots differing in their embodiment and degree of realism to check whether our findings still hold. We also suggest to replicate our study involving a larger and more diverse set of participants, particularly when it comes to academic background and gender.

Unlike most related work (e.g., Hofree et al., 2014), in our experiment, we included stimuli covering all six basic emotions (Ekman et al., 2002). For the analyses of facial mimicry, however, we combined people’s responses to the different emotions together and calculated an average facial mimicry value. Especially given the differences observed between individual expressions in our validation study, it is fair to assume that, while comprehensive, our results might not fit all six basic emotions equally. Another element of variation that one might need to control when studying facial mimicry is the observer’s belief that an agent’s facial expression reflects its subjective emotional state. In future facial mimicry studies, it would be interesting to include additional questionnaires capturing people’s belief about the agent’s emotional state when performing facial expressions, and their own emotional state before and after the experiment. While a mixed-measure experimental design has its advantages especially in very controlled environments that make recruiting a large group of participants difficult, we acknowledge that the repeated measures in the current study design was probably tiring for participants and may have lead to them paying less attention to the stimuli they encountered as last. While we randomized the order of presentation of the different conditions to control for any order effect, it would overall be beneficial to replicate this experiment with a between-subject design., especially if potentially expanding the set of questionnaires to be included in the study.

Since our study was task-based, non-interactive, and devoid on an emotional context, the acted nature of the agents’ facial expressions was particularly clear. Future work should focus on bringing the study of facial mimicry into more interactive and social contexts and assess whether facial mimicry could be used in place of questionnaires to assess people’s social attunement with artificial agents. An important pre-condition for using facial mimicry as a behavioral indicator of people’s relationship with a robot is a robust and non-intrusive assessment technique of people’s facial expressions. While the computer-vision-based approach discussed in this paper has shown promising results, further improvements are necessary to make it more robust with respect to different angles and light conditions. This is especially important if we want to bring the study of facial mimicry to less controlled scenarios.

## 9 Conclusion

In the study presented in this paper, we involved participants in an emotion recognition task carried out with artificial agents differing in their embodiment and degree of humanlikeness. In the first phase of the study, we asked participants to observe the artificial agents’ facial expressions and attempt to identify the emotions they displayed. In the second phase of the study, instead, we asked participants to observe the agents’ facial expressions, mimic them as closely as possible, and then identify them. We used the first part of the study to investigate the frequency of participants’ spontaneous facial mimicry, and the second part to investigate the accuracy of their instructed facial mimicry. The aim was to understand whether spontaneous mimicry of artificial agents’ facial expressions can be used as a behavioral cue of liking and rapport, and whether instructed facial mimicry could act as a proxy of its spontaneous counterpart. Our results suggest that, in an emotion recognition task, the physical instantiation of an artificial agent, together with its likability and anthropomorphism, intrudes rather than promotes people’s spontaneous facial mimicry. Furthermore, results suggest that instructed facial mimicry negatively predicts spontaneous facial mimicry. Since the participants in this study mimicked the facial expressions of the artificial agents more when they were uncertain about the emotion to recognize, one possibility is that, in emotion recognition contexts, facial mimicry serves the purpose of emotion recognition. Even though our results did not support our initial hypotheses, they nevertheless show that spontaneous mimicry can be a behavioral cue of liking and rapport, and instructed facial mimicry a proxy of spontaneous facial mimicry.

## Data Availability

The dataset presented in this article is not readily available because it contains personal information on the participants. An anonymized version of the data supporting the conclusions of this article can be made available by the authors upon request.

## Footnotes

1
https://facegen.com/modeller.htm

2
https://ffmpeg.org/
